# Investigation of Hypodontia as Clinically Related Dental Anomaly: Prevalence and Characteristics

**DOI:** 10.5402/2011/246135

**Published:** 2010-09-29

**Authors:** Young Ho Kim

**Affiliations:** Professor and Chairman, Department of Orthodontics, The Institute of Oral Health Science, Samsung Medical Center, Sungkyunkwan University School of Medicine, 50 Irwon-dong, Gangnam-Gu, Seoul 135-710, Republic of Korea

## Abstract

*Objective*. Patients with hypodontia are relatively common in clinical dentistry. This study was performed to determine the prevalence of hypodontia of permanent teeth in Korean orthodontic patients and whether such prevalence is associated with the type of dental clinic, patient gender, or the type of malocclusion. *Materials and Methods*. Over a five-year period, we evaluated 3,055 patients (mean age, 15.1 years; range 9~30) from two geographically separated orthodontic clinics: 1,479 from University Hospital and 1,576 from a private clinic. Hypodontia was diagnosed using panoramic radiographs, clinical examination, and dental casts. *Results*. The overall prevalence of hypodontia, excluding the third molars, was 11.3%, and there was no statistically significant association with the type of dental clinic, gender, or malocclusion patterns. The most commonly missing teeth were the mandibular second premolars (44.2%), followed by the mandibular lateral incisors (36.6%), and the maxillary second premolars (34.0%). In both sexes, 86.0% of patients with hypodontia were missing one or two teeth. *Conclusion*. The relatively high prevalence of hypodontia emphasizes the importance of dental examination in early childhood with radiographic screening for hypodontia as standard public oral health policy and warrants further investigation of the orthodontic treatment strategies to prevent resultant oral health impairments of hypodontia.

## 1. Introduction

Clinicians often encounter patients with hypodontia, which usually causes oral health impairment. The reported prevalence of hypodontia of permanent teeth varies from 0.3% [1] to 36.5% [2]. Accurate diagnosis of hypodontia requires radiographic, clinical, and dental cast examinations to distinguish whether the tooth is extracted, impacted, or congenitally absent. It is often difficult to accurately distinguish the missing tooth from adjacent similar tooth. For example, it can be hard to distinguish between the mandibular central incisor and lateral incisor when there are three incisors. For those cases, meticulous examination of dental casts is helpful. Although studies on hypodontia have been performed previously, some of these were limited by the lack of thorough radiographic examinations [3, 4] or small sample size [5–7], because a mass survey with radiographic and dental cast examinations for the general population is extremely expensive to perform. Furthermore, most of all researches included subjects under eight of age. In general, visual inspection or radiographic diagnosis of missing permanent posterior teeth cannot be guaranteed until the age of nine [8]. 

Variation in the distribution and location of hypodontia has been reported across ethnic groups. Notably, a higher incidence of missing mandibular incisors is observed in Chinese [9, 10] and Japanese populations [11, 12] than in Caucasian populations, [8, 13, 14] suggesting that ethnicity factors into prevalence. Although the prevalence of hypodontia in patients with specific congenital facial dysplasia (cleft lip and palate) has been reported in Koreans [15], the prevalence in the general population is not known.

In order to estimate the prevalence of hypodontia, we evaluated patients who visited two geographically different orthodontic clinics; a private local dental clinic in the country and a university affiliated general hospital in the principal town. Factors that may influence the prevalence of hypodontia, including the difference between the type of dental clinic, gender, age, and the type of malocclusion are also discussed.

## 2. Materials and Methods

### 2.1. Subjects

The inspection module was based on a five-year period of total subjects inspection between 2004 and 2009. A Poisson sampling model, without fixing the total sample size, was applied. A total of 3,055 subjects from two geographically distinct orthodontic clinics were examined using panoramic radiograph, periapical radiograph for the incisor region, and dental casts. In cases where it was difficult to distinguish between extraction and hypodontia, the previous dental history was examined. 

Patients aged from nine to 30 years were included to take into account the late onset of mineralization of mandibular second premolars. Third molars were not included in this study. Patients with developmental anomalies including oral clefts and systemic diseases were excluded from this study. Two patients with developmental disease, one organic disease and one ectodermal dysplasia, were also excluded. 

The two investigation centers, selected in this study, had similar number of orthodontic patients during a five-year period. A total of 1,576 subjects were obtained from a private orthodontic clinic located in a rural middle-class area in Gyeonggi-do and 1,479 subjects were obtained from an orthodontic clinic in university affiliated general hospital located in a metropolitan middle-class area in South-Eastern Seoul (Table 1).

### 2.2. Statistical Data Analysis

All data were recorded using Microsoft Excel worksheets and analyzed by statistical software (SPSS 12.0, Chicago, Ill). To test data quality, 10% of the data were randomly selected and reevaluated by an investigator (YH-K) one month after the initial examination so that 100% reproducibility was assured in identification and localization of congenital missing teeth. A Cochran-Mantel-Haenszel test was used to determine whether the prevalence of hypodontia is significantly different according to gender and malocclusion class after controlling for the characteristics of two investigation centers. For this purpose, the investigation center was dealt as a control or stratification variable into the computation of the odds statistics. The null hypothesis was that the prevalence of hypodontia is independent in the two centers. Because the prevalence across the two centers was approximately equal, the data were combined into a pooled estimate of a common odds ratio. In addition, homogeneity between two centers was tested using the Breslow-Day test. For all inferential tests, a probability of less than .05 was considered statistically significant.

## 3. Results

The subjects were composed of 39.4% male and 60.6% female patients with no difference in gender distribution between the private local clinic and the university affiliated general hospital (Table 1). The mean age was 15.1 years, ranging from nine to 30 years.

The overall prevalence of hypodontia of permanent teeth, excluding the third molars, was 11.3%. The prevalence of hypodontia was 10.3% for the private local clinic and 12.2% for the university affiliated general hospital (Table 2). However, the difference between the two investigation centers was not statistically significant (95% CI for odds ratios: 0.966, 1.513).

The prevalence of hypodontia according to gender was 9.5% in males and 12.4% in females (odds ratios 1.342; 95% CI: 1.06, 1.697) (Table 2). However, because the odds ratio and 95% CI was close to 1.0, the difference in the prevalence of hypodontia between genders was clinically not significant. Furthermore, there was no indication that the odds ratios differed between gender groups (Breslow-Day test for homogeneity across the two investigation centers *P* = .515). 

The prevalence of hypodontia according to the age subgroups 9–19 years and 20~30 years was 10.6% and 11.1%, respectively, (Table 2). The reason to divide those subgroups was simply to estimate the difference between the hypodontia in adolescent and young age group and that in adult group. The odds ratio was close to 1.0 and the 95% CI indicating that the difference in the prevalence of hypodontia between the age subgroups was not statistically significant. 

Even though the prevalence of hypodontia in Angle Class II division 2 was higher (16.7%) than that in other Angle classifications (10.2%–12.5%), the sample size of the Angle Class II division 2 was too small (72 out of total 3055 subjects) and the prevalence of hypodontia was not significantly different according to the type of malocclusion (Table 2).

The frequency and percentage of hypodontia with respect to the number of missing teeth in both sexes combined showed that 50.0% of patients with hypodontia were missing one tooth, 36.0% missing two, 7.0% missing three, 3.2% missing four, 2.9% missing five, and 0.9% missing more than six teeth (Table 3). Of the total samples studied, three patients (0.098%) were found to miss six or more teeth, consistent with oligodontia.

**Figure 1:**
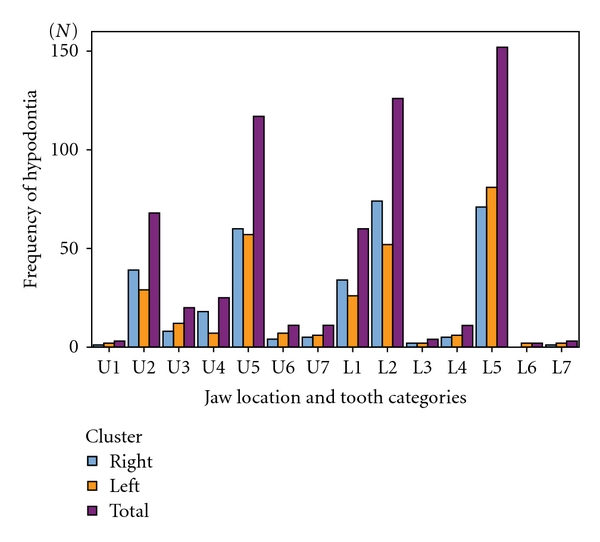
Frequency and percentage of hypodontia with respect to jaw location and tooth category.

The most commonly missing teeth were the mandibular second premolars (44.2%), followed by the mandibular lateral incisors (36.6%), the maxillary second premolars (34.0%), the maxillary lateral incisors (19.8%), and the mandibular central incisors (17.4%) (Figure 1). The frequency of hypodontia in the mandible (225) was higher than that in the maxilla (166). The frequency of unilaterally missing teeth (182) was not different from that of bilaterally missing teeth (162). In contrast, symmetric hypodontia (111) was more predominant than asymmetric hypodontia (51) in bilaterally missing teeth.

## 4. Discussion

The prevalence of hypodontia was higher in the orthodontic clinic of university affiliated general hospital (12.2%) than that in private local clinic (10.3%), but it was not statistically significant. Regardless of the type of dental clinic, these samples were highly selected individuals with malocclusion, probably referred to these clinics by other dental health professionals. It was likely that they also had been referred on ground congenital missing teeth and not on the existence of malocclusion only. From this assumption, the prevalence of hypodontia in the general population of Korea would probably be somewhat lower than the result of this study, even though the fact that the prevalence of hypodontia was not different between the two dental clinics might suggest that patients from the two centers could have similar dental characteristics.

Even though there was no statistically significant difference in the prevalence of hypodontia between genders, female (12.4%) had higher prevalence rate than male (9.5%). At a glance, this difference was not small, suggesting that females might have more predominance tendency on the prevalence of hypodontia. The result of no gender difference was in agreement with the results of several previous reports [4, 12, 13, 16–18], although other studies report female predominance with respect to the prevalence of hypodontia [8, 14, 19–22]. 

One critical issue in a study on hypodontia is the age at diagnosis, since visibility of tooth germs on radiographs depends on the stage of mineralization [14]. The stages of dental development are more closely related to mineralization of the teeth than is chronologic age [19]. Therefore, the age ranges of the patients were selected to take into consideration the late development of the mandibular second premolars in boys [8] and DS 3 (canines or premolars erupting) or DS 4 stages (canines and premolars fully erupted) of dental development according to the classification of Dental Stage (DS) developed by Bjoerk et al. [23] DS 1 (incisors erupting), DS 2 (incisors fully erupted), and earlier stages were not included. 

Although the age ranges were divided into 2 subgroups (9–19 years and 20–30 years) in the results presented, there were no significant differences in the prevalence of hypodontia when the samples were divided into 3 subgroups (9–14, 15–19, and 20–30 years) or 4 subgroups (9-10, 11–14, 15–19, and 20–30 years). 

The prevalence of malocclusion based on Angle's classification showed a marked increase in the number of Class II (36.12%) and Class III (31.7%) patients, compared with the proportion of Class II (20.3%) and Class III (19.0%) in the Korean general population [24]. This may represent the dental characteristics of the patients who visited the orthodontic clinic for the treatment of malocclusion. Even though the results that the prevalence of hypodontia was not associated with the type of malocclusion might indicate that congenital missing teeth may not affect the prevalence of malocclusion, these samples were orthodontically selected material, probably referred for orthodontic treatment, and some controversies may remain. However, Angle Class II division 2 had a high prevalence of hypodontia compared with the other categories of malocclusion, though a very small subsampling of missing teeth in 12 individuals. If the sample size of the Class II division 2 could be increased near the size of the other categories, the results might be changed. This result was in agreement with a previous report [25] showing no relationship between hypodontia and Class III, or Class II division 1 malocclusion. But in the case of the prevalence of Class II division 2, further investigation was needed with considering previous reports that hypodontia was closely associated with Class II division 2 malocclusion [26] and Class II malocclusion [27]. 

The most commonly missing permanent teeth were the mandibular second premolars, which is in accordance with many previous reports [8, 12–14, 16, 17, 19–21, 28], but differs from other reports in which the most commonly missing teeth are the maxillary lateral incisors [4, 18, 22, 27]. The prevalence of missing mandibular lateral and central incisors was 54.0% in our Korean population, comparable to values of 60.2% in Chinese [9] and 47% in Japanese [11]. The prevalence of missing mandibular lateral incisors and mandibular central incisors was 36.6% and 17.4%, respectively, markedly different from corresponding levels of 7.3% and 10.2% reported in Caucasian populations [13]. The high prevalence of the absence of mandibular incisors, particularly the mandibular lateral incisors, may be a major characteristic of hypodontia of “mongoloids,” one of the major human racial groups, distributed widely through Asia from the Caspian sea eastwards. These results were in accordance with some previous reports about the Japanese, Chinese, and Eskimos [9, 11, 12]. 

Analysis with respect to the number of congenitally missing teeth showed that in both sexes 86.0% of patients were missing one or two teeth; this proportion is within the range of 75.0% to 90.9% obtained from many previous studies [8, 12, 14, 17], with the exception of one extremely high report of 97.4% [9] and one extremely low report of 49.0% [13]. 

The higher frequency of hypodontia in mandible than in maxilla was in agreement with some reports [8, 16, 17], contrary to the reports of maxillary predominance [18, 22, 27], and other report which shows no significant difference [12]. The frequency of unilaterally missing teeth was not different than that of bilaterally missing teeth. This is in contrast to the report on the predominance of bilaterally missing teeth [13] and the report on the predominance of unilaterally missing teeth [8, 14]. In bilaterally missing teeth, symmetric hypodontia was more predominant than asymmetric hypodontia, which is in agreement with previous reports [18, 19, 22]. 

Even though hypodontia has been diagnosed more often in recent studies [29], the actual trend of tooth loss throughout the evolution of mankind is unknown. However, the current trend of hypodontia that occurs frequently at the integrated portion of anterior, premolar and molar teeth (second premolar and lateral incisor) appears to minimize disruption of their functional integrity. This is similar to previous findings in primates [30], in which the decrease in the number of incisors parallels the process of regression of mastication during evolution. 

Hypodontia is the most prevalent dental anomaly in children [31] and its prevalence in this study was higher than that of diabetes, which is a widely known disease with a reported prevalence of 7.6% [32] in Korea. However, the clinical importance of hypodontia is not recognized by the general population.

The orthodontic treatment strategies for such high prevalence and diverse patterns of hypodontia are needed to prevent oral health impairment. In cases of missing mandibular incisors especially require the functional and esthetic camouflage on the relationship between maxillary and mandibular anterior teeth. In replacing congenitally missing teeth also may consider the bone volume, which is related to the facial esthetics including smile [33]. The multidisciplinary approaches for the care of the hypodontia patients are also important to consider the impact of hypodontia on the quality of life [34], and the establishment of the “Hypodontia Clinic” in the University Hospital is recommended for the total care of hypodontia patients, who have most common complaints of missing teeth, spacing in the dental arches, and poor appearance [35].

## 5. Conclusions

The prevalence of hypodontia of permanent teeth in Koreans, excluding third molars, was 11.3%, more than one in ten. The most commonly missing teeth were the mandibular second premolars, followed by the mandibular lateral incisors, and the maxillary second premolars. The prevalence of hypodontia in Koreans is relatively high, and dental examination with radiographic screening of hypodontia in early childhood should be emphasized as part of public oral health policy. The results of this study warrant further investigation of the orthodontic treatment strategies to prevent resultant oral health impairments of hypodontia.

## Figures and Tables

**Table 1:** Distribution of subjects by gender and the type of dental clinic.

Gender	Investigation centers		Total
	Private local clinic	University affiliated general hospital	
Male	615 (39.0%)	590 (39.9%)	1,205 (39.4%)
Female	961 (61.0%)	889 (60.1%)	1,850 (60.6%)
Total	1,576 (100%)	1,479 (100%)	3,055 (100%)

**Table 2:** Prevalence of hypodontia according to the type of dental clinic, gender, age subgroups, and the type of malocclusion.

Variables (Total *N* = 3,055)	*N* (%)		Odds ratio*	95% CI	*P*-value^†^
	Normal	Hypodontia			
Hospital characteristics					
Private local clinic	1413 (89.7%)	163 (10.3%)	1.212	0.966, 1.513	.515^†^
University hospital	1,298 (87.8%)	181 (12.2%)			

Gender					
Male	1,090 (90.5%)	115 (9.5%)	1.342	1.06, 1.697	.515^†^
Female	1,621 (87.6%)	229 (12.4%)			

Age subgroups					
9~19	1,880 (89.4%)	224 (10.6%)	1.172	0.919, 1.494	.201^†^
20~30	788 (88.9%)	110 (11.1%)			

Malocclusion classification					.163^‡^
Class I	862 (87.5%)	123 (12.5%)			
Class II division 1	925 (89.8%)	105 (10.2%)			
Class II division 2	60 (83.3%)	12 (16.7%)			
Class III	864 (89.3%)	104 (10.7%)			

Total	2711 (88.7%)	344 (11.3%)			

*Mantel-Haenszel common odds ratio estimates. ^†^Homogeneity of odds ratio was tested using the Breslow-Day test. ^‡^Difference of prevalence among malocclusion classifications was tested using Fisher's exact test.

**Table 3:** Frequency and percentage of hypodontia with respect to the number of missing teeth.

	Number of missing teeth						
	1	2	3	4	5	≥6	Total
Frequency	172	124	24	11	10	3	344
Percent	50.0	36.0	7.0	3.2	2.9	0.9	100.0
